# Targeting the PI3K/Akt/mTOR Pathway in Hepatocellular Carcinoma

**DOI:** 10.3390/biomedicines9111639

**Published:** 2021-11-08

**Authors:** Eun Jin Sun, Miriam Wankell, Pranavan Palamuthusingam, Craig McFarlane, Lionel Hebbard

**Affiliations:** 1 Centre for Molecular Therapeutics, Department of Molecular and Cell Biology, Australian Institute of Tropical Medicine and Health, College of Public Health, Medical and Veterinary Sciences, James Cook University, Townsville, QLD 4811, Australia; eunjin.sun@my.jcu.edu.au (E.J.S.); miriam.wankell@jcu.edu.au (M.W.); craig.mcfarlane@jcu.edu.au (C.M.); 2 College of Medicine and Dentistry, James Cook University, Townsville, QLD 4811, Australia; 3 Institute of Surgery, The Townsville University Hospital, Townsville, QLD 4811, Australia; Pranavan.Palamuthusingam@health.qld.gov.au; 4 Mater Hospital, Townsville, QLD 4811, Australia; 5 Storr Liver Centre, Westmead Institute for Medical Research, Westmead Hospital and University of Sydney, Sydney, NSW 2145, Australia

**Keywords:** hepatocellular carcinoma, HCC, Akt, mTOR, PI3K, clinical trials

## Abstract

Despite advances in the treatment of cancers through surgical procedures and new pharmaceuticals, the treatment of hepatocellular carcinoma (HCC) remains challenging as reflected by low survival rates. The PI3K/Akt/mTOR pathway is an important signaling mechanism that regulates the cell cycle, proliferation, apoptosis, and metabolism. Importantly, deregulation of the PI3K/Akt/mTOR pathway leading to activation is common in HCC and is hence the subject of intense investigation and the focus of current therapeutics. In this review article, we consider the role of this pathway in the pathogenesis of HCC, focusing on its downstream effectors such as glycogen synthase kinase-3 (GSK-3), cAMP-response element-binding protein (CREB), forkhead box O protein (FOXO), murine double minute 2 (MDM2), p53, and nuclear factor-κB (NF-κB), and the cellular processes of lipogenesis and autophagy. In addition, we provide an update on the current ongoing clinical development of agents targeting this pathway for HCC treatments.

## 1. Introduction

Cancer of the liver is represented by cholangiocarcinoma and hepatocellular carcinoma (HCC). HCC constitutes approximately 80–90% of all primary liver cancers and has a high mortality rate while many are detected in later stages of development and therapeutic options are limited [1]. Worldwide, HCC is now the sixth most common cancer and the fourth leading cause of cancer-related death [2,3,4]. The major known causes of HCC are alcohol abuse, diabetes, obesity, hepatitis B virus (HBV) and hepatitis C virus (HCV) infections, aflatoxin B1, and non-alcoholic fatty liver disease (NAFLD) and its progressed form non-alcoholic steatohepatitis (NASH), which was recently renamed metabolic associated fatty liver disease (MAFLD) [5,6,7]. Together, these promote hepatic inflammation that can progress to fibrosis and cirrhosis, and in turn hepatocyte apoptosis and oxidative stress, leading to altered protein expression, DNA damage, and carcinogenesis. In this regard, formative studies noted mutations and dysregulated expression of the phosphatidylinositol 3-kinase (PI3K)/the serine-threonine protein kinase (Akt)/mammalian target of the rapamycin (mTOR) signaling pathway in HCC. This encouraged the application of small molecule inhibitors of the PI3K/Akt/mTOR pathway in pre-clinical models and subsequent treatment of patients [8,9,10,11,12,13]. Considering HCC incidence is rapidly increasing worldwide and therapeutic choices are limited, here we consider the role of the PI3K/Akt/mTOR pathway in HCC, while it is a focus of current clinical practice and subject of intense investigation for future therapeutic development.

## 2. HCC Drivers and Outlook

Since 1990, there has been a 114% increase in world-wide liver cancer incidence with significant rises in countries with high socio-demographic indexes, particularly in the USA, Australia, the UK, and the Netherlands [14,15]. This has occurred despite the introduction of HBV vaccines in the 1990s and HCV anti-viral therapies in the last decade. These initiatives decreased the rates of HBV- and HCV-driven HCC in developed countries, and these HCC forms are becoming a disease of underdeveloped countries [2,3,4]. In contrast, with increasing rates of alcohol abuse and NAFLD/MAFLD, the expectation is that HCC incidence will increase in developed countries [16,17,18]. NAFLD/MAFLD represents conditions ranging in severity from simple steatosis, non-alcoholic steatohepatitis (NASH), to advanced fibrosis and cirrhosis [16]. Recent analyses show the overall global prevalence of NAFLD/MAFLD diagnosis to be in the vicinity of 25%, and it commonly associates with comorbidities such as obesity, diabetes mellitus, and the metabolic syndrome [16,17]. In the US, NAFLD and NASH cases are estimated to increase 21% and 63%, respectively, by 2030 and this is expected to coincide with increased HCC incidence [19].

## 3. Current Treatments of HCC

Treatment options for early to intermediate stages of HCC remain relatively unchanged, including surgical resection, percutaneous ethanol injection (PEI), radiofrequency ablation (RFA), trans-arterial chemoembolization (TACE), and liver transplantation. However, these procedures are only suitable for a small number of patients, mostly those without cirrhosis, and often have post-operative complications [20]. The introduction of Sorafenib, a multi-kinase inhibitor in 2007, has significantly altered the treatment for intermediate and advanced stage HCC [13]. In advancing intermediate HCCs, the combination of TACE and pharmaceutical agents are frequently used, as this is more effective than monotherapy [21]. Importantly, with the approval of additional drugs, the current algorithm of treatment for advanced HCC includes sorafenib and lenvatinib as first-line and regorafenib, cabozantinib, ramucirumab, nivolumab, and pembrolizumab as second-line add-on pharmaceuticals [22]. Sorafenib, regorafenib, lenvatinib, and cabozantinib are multitargeted tyrosine kinase inhibitors (TKI) that target tumor cell proliferation and angiogenesis pathways. Sorafenib and regorafenib share a common targeting profile as regorafenib is a derivative of sorafenib. Sorafenib targets tyrosine kinase signaling including rapidly accelerated fibrosarcoma (RAF), vascular endothelial growth factor (VEGF) receptors 1–3, and platelet-derived growth factor (PDGF) receptor β, and regorafenib additionally blocks v-raf murine sarcoma viral oncogene homolog B1 (B-RAF), proto-oncogene c-KIT (KIT), ret proto-oncogene (RET), angiotensin 1 receptor (TIE2), PDGFRα, and fibroblast growth receptors (FGFRs) 1 and 2 [23,24]. Lenvatinib is a tyrosine kinase inhibitor that inhibits angiogenesis by targeting VEGFR1-3, fibroblast growth factor receptor (FGFR) 1–4, PDGFR, and tumor cell expressed RET and KIT. Cabozantinib similarly targets the aforementioned proteins, and as well MET (hepatocyte growth factor receptor), neurotrophic receptor kinase 2 (NTRK2), Fms Related Receptor Tyrosine Kinase 3 (FLT3), and AXL receptor tyrosine kinase (AXL). Ramucirumab is a direct binding VEGFR2 antagonist and blocks the binding of VEGF ligands -A, -C, and -D. Nivolumab and pembrolizumab are immune check-point inhibitors and will be discussed in later sections.

Despite sorafenib’s usage as a first-line treatment option for advanced HCC, fewer than one-third of patients benefit from treatment and have just three months prolonged median survival time [13,25]. Moreover, the use of chemotherapeutic agents can lead to drug resistance within six months of initiation and complications such as drug inefficacy leading to increased dosage and toxicity [25]. This is of concern as the current available first-line drugs are TKIs that target the PI3K/Akt/mTOR pathway. Hence, in this review we will consider the role of the PI3K/Akt/mTOR pathway in HCC, the efficacy of current TKI drugs, and their application in new therapeutic approaches.

## 4. The PI3K/Akt/mTOR Pathway

The PI3K/Akt/mTOR pathway is a major intracellular signal transduction pathway involved in regulating the cell cycle, cell proliferation, apoptosis, metabolism, and angiogenesis through communicating with its related upstream and downstream molecules and is activated in many cancer types through the action of dysregulated receptor tyrosine kinases (RTK) [26,27]. RTK monomers are high-affinity cell surface receptors for growth factors, cytokines, and hormones that, on ligand binding, become activated and dimerize, causing each monomer to autophosphorylate, leading to downstream activation of the PI3K/Akt/mTOR pathway [28,29,30]. Activated RTKs recruit PI3K that phosphorylates phosphatidylinositol on the internal plasma membrane. PI3K is classified into three classes (I, II, and III) according to their structure and substrate specificity, and class IA PI3Ks is the main enzyme that associates with oncogenesis. PI3K can be directly activated by regulatory subunits binding to RTKs and GTP-binding rat sarcoma virus (RAS), or indirectly activated by adaptor molecules such as the insulin receptor substrates (IRS).

Active PI3K phosphorylates phosphatidylinositol-4,5-bisphosphate (PIP2) to phosphatidylinositol-3,4,5-triphosphate (PIP3) [31,32]. PIP3 can interact and recruit 3-phosphoinositide dependent protein kinase 1 (PDK1) and Akt to the plasma membrane, and PDK1 phosphorylates Thr308 of the activation loop to partially activate Akt [33,34]. Akt, also known as protein kinase B (PKB), is a serine/threonine-specific protein kinase that has an integral role in various cellular mechanisms [35]. Akt consists as three isoforms: Akt1, Akt2, and Akt3, and they are expressed in different tissues within the human body. Akt1 is widely expressed, Akt2 is mainly expressed in insulin-sensitive tissues, and Akt3 is expressed in the brain and testis. The major difference among these three subtypes are the functional domains that regulate various downstream protein–protein and protein–lipid interactions. Phosphorylation of both Thr308 and Ser473 located in the regulatory region of Akt, respectively by PDK1 and PDK2, are required to complete Akt activation [35]. Akt is also regulated by the tumor suppressor Phosphate and Tensin Homolog (PTEN), a phosphatase that dephosphorylates PIP3 to produce PIP2. Complete Akt activation stimulates targets downstream proteins, including mammalian target of rapamycin (mTOR), glycogen synthase kinase-3 (GSK-3), cAMP-response element binding-protein (CREB), forkhead box O protein (FOXO), p53 and nuclear factor-kβ (NF-kB), and cellular processes including lipid metabolism and autophagy.

### 4.1. mTOR

mTOR is a serine/threonine protein kinase that is part of the PI3K-associated kinase protein family and mTOR’s major cellular role is to regulate cell growth and proliferation through nutritional signals. mTOR is present in two cellular complexes: mTOR complex 1 (mTORC1) and complex 2 (mTORC2). mTORC1 is composed of mTOR, the regulator-associated protein of mTOR (Raptor), while mTORC2 contains rapamycin-insensitive companion of mTOR (Rictor). Together these proteins act as a framework for assembling the complexes and binding substrates. DEP domain-containing mTOR interacting protein (DEPTOR) and the Mammalian lethal with SEC13 protein 8 (mLST8) associates with both mTORC1 and mTORC2. In addition, mTORC2 contains protein observed with rictor-1 (Protor-1), Protor-2, and mammalian stress-activated protein kinase-interacting protein 1 (mSIN1). Both mTOR complexes also bind with a number of inhibitor proteins that regulate their activity; mTORC1: proline rich Akt substrate 40 (PRAS40) and FKBP38; and mTORC2: exchange factor found in platelet, leukemic, and neuronal tissues (XPLN) that can negatively regulate mTORC2. mTORC1 regulates cell growth and energy metabolism whereas mTORC2 is involved in the reconstruction of cytoskeletons and regulation of cell survival.

As a PDK2, mTORC2 phosphorylates Akt on Ser473 to fully activate Akt, that can then in turn activate mTORC1. In addition to phosphorylated Akt, Ras homolog mTORC1 binding (Rheb), a small GTPase, is required for mTORC1 activation. Rheb is normally inhibited by tuberous sclerosis complex subunit 2 (TSC1/2) a GTPase-activating protein (GAP) inhibitor. Akt can inhibit TSC1/2 leading to Rheb and in turn mTOR activation in mTORC1. The downstream effectors of mTORC1 include the eukaryotic translation initiation factor 4E binding protein 1 (4EBP1) and ribosomal proteins S6 kinase 1 and 2 (S6K1/2), which are involved in mitochondrial biosynthesis and regulation of mRNA translation. Given these integral roles, the dysregulation of both mTORC1 and mTORC2 has been observed in many human solid tumors. Studies have also shown that mTOR inhibition associates with autophagy activation, and in contrast mTOR hyperactivation can increase lipid production that parallels obesity, diabetes, and fatty liver disease [36,37,38].

### 4.2. Glycogen Synthase Kinase-3 (GSK-3)

GSK-3 a well-known substrate of Akt, is an ubiquitously expressed serine/threonine protein kinase that exists as two isoforms, GSK-3α and GSK-3β with 85% homology [39]. They are constitutively active and inhibit glycogen synthase, the key enzyme in glycogen synthesis. In response to insulin receptor activation, Akt is phosphorylated and in turn phosphorylates and inhibits GSK-3α on Ser21 and Tyr279 and GSK-3β on Ser9 and Tyr21 [40,41]. GSK-3 controls a range of downstream substrates, including c-Myc, hypoxia inducible transcription factors 1α (HIF-1α), sterol regulatory element binding protein 1c (SREBP1c), and forkhead/winged helix family k1 (Foxk1). Phosphorylation of GSK-3β also leads to the accumulation of β-catenin within cells and translocation to the nucleus to promote T-cell factor (TCF) transcription targets, such as c-Myc, c-Jun, and cyclin D1 [42]. mTORC1 can also directly modulate GSK-3 activity by restricting GSK3-mediated phosphorylation of Foxk1. This causes the nuclear accumulation of Foxk1 and binding to promoters of several metabolic genes such as those involved in glucose metabolism and HIF-1α to induce cell growth and tumor development [43]. Reports have shown that GSK-3β upregulation in HCC predicts poor patient prognosis and that GSK-3β inhibition with short-hairpin RNA (shRNA) or specific inhibitors can decrease mTORC1 activity, glycolysis, and HCC growth in vivo [44]. These data illustrate an important role for GSK-3β in HCC.

### 4.3. cAMP-Response Element-Binding Protein (CREB)

Activity of PI3K and Akt also leads to the phosphorylation of the transcription factor cyclic adenosine 3′,5′-monophosphoate (cAMP)-response element binding-protein (CREB) on Ser133 to cause CREB dimerization and activation [45]. CREB binds the cAMP response element (CRE) of gene promoters, is expressed in all nucleated cells, and associates with expression of genes that control proliferation, apoptosis, angiogenesis, metastasis, and metabolism. Additionally, GSK-3β can phosphorylate CREB on Ser129 to enhance activity [46]. Therefore, inhibiting Akt and/or GSK-3β can limit CREB activity and gene activation. CREB expression is also positively regulated by TSC2 through mTOR, and the dysregulation of mTOR or loss of TSC2 can lead to the overexpression or hyperactivation of CREB, disrupting autophagy regulation. Furthermore, studies show the association of activated CREB with increased HCC invasion and hence poor prognosis [47], and compared to the normal liver, total and phosphorylated CREB proteins are significantly increased in HCC [48]. In addition, under hypoxic conditions, the knockdown of CREB reduced HCC proliferation and limited angiogenesis and made HCC cells susceptible to chemotherapy in vitro and in vivo [49]. In contrast, the over-expression of a positive-dominant CREB mutant supported HCC growth, angiogenesis, and resistance to apoptosis [50]. CREB overexpression also restored the expression of the oncogene Yes-associated protein (YAP) in HCC cells to promote HCC proliferation, and the HBV X protein can enhance this association in HBV-driven HCC [51,52]. Other reports have illustrated that decoy oligonucleotides or p38 MAPK inhibitors promote CREB degradation and the radiosensitivity of HCC cells, suggesting a role for CREB in radiotherapy prognosis in HCC patients [53].

### 4.4. Forkhead Box O Protein (FOXO)

The forkhead box O protein (FOXO) is a transcription factor and consists of four members: FOXO1, FOXO3, FOXO4, and FOXO6. Akt-mediated phosphorylation of FOXO inhibits their function by promoting their export from the nucleus to the cytoplasm. FOXO1 is expressed in the liver, pancreas, fat, and muscle tissues, whereas FOXO3 and FOXO4 are expressed in the lymph node, liver, kidney, heart, and skeletal muscles [54]. FOXO6 is mainly expressed in the nervous system, including the brain. FOXOs function by integrating signals such as growth factors, oxidative stress, and other stimulatory signals to induce gene expression of downstream targets involved in cell cycle, metabolism, apoptosis, DNA damage, oxidative stress, and stem cell differentiation. An important regulator of FOXO is insulin signaling, which through Akt leads to FOXO1 and FOXO3 phosphorylation, binding to 14-3-3 proteins, translocation to the cytoplasm, and subsequent dissociation from 14-3-3 for ubiquitination and degradation, to inhibit FOXO-mediated transcription [55,56].

FOXO1 and FOXO3 are the main FOXO proteins that contribute to HCC tumorigenesis and progression [55]. In HCC, hyperactive Akt signaling inhibits FOXO1 transcriptional activity, weakens defense against oxidative stress, and as FOXO1 normally suppresses the expression of epithelial mesenchymal transition (EMT)-inducing transcription factors and transforming growth factor-β (TGF-β), leads to subsequent EMT and increased HCC cell migration and invasion [57]. In contrast, reports have established an association between high FOXO3 expression and poor prognosis. In the normal liver, FOXO3 expression is limited, whereas FOXO3 overexpression is observed in more than half of HCCs and correlates with poor disease-free survival and prognosis [58,59]. Furthermore, the doxycycline-regulated over-expression of FOXO3 in the murine liver supported hepatotoxicity-mediated HCC growth, the accumulation and elimination of reactive oxidative species (ROS), and the activation of Akt and mTORC2 signaling [60]. With regards to sorafenib treatment, FOXO3 ablation inhibited sorafenib-induced autophagy and increased cytotoxicity to sorafenib in HCC cells and xenograft tumors, suggesting the development of FOXO3 targeted therapy may be a promising approach to augment sorafenib action [61].

### 4.5. Murine Double Minute 2/Human Double Minute2 (MDM2/HDM2)–p53 Axis

The transcriptional factor p53 acts as a tumor suppressor by modulating apoptosis, cell cycle arrest, and senescence, and is upregulated in response to cellular stresses such as DNA damage, hypoxia, and nutritional starvation [62]. p53 is one of the most often mutated genes in all human cancers as either a gain or loss of function, and contributes to increased proliferation, survival, EMT, and metastasis. p53 mutations are readily observed in HCC with a reported range of 22 to 33% [63]. However, this varies between geographical regions and presence of hepatitis virus and carcinogen exposure, in particular the combination of HBV and aflatoxin B1 exposure where a specific p53 mutation is detected in more than 75% of HCCs [64].

Normally, p53 protein expression is regulated by the E3 ubiquitin ligase murine double minute 2 (MDM2) by binding to p53 to restrict p53 mediated transcriptional activation, and promote p53 translocation to the cytoplasm for proteosome-dependent degradation via ubiquitination [62]. In turn, MDM2 is a transcriptional target of p53 and MDM2-p53 homeostasis is maintained through an auto-regulatory negative feedback loop as p53 enhances MDM2 transcription and MDM2 downregulates and degrades p53 in response to excess p53. Additionally, Akt can regulate p53 stability by phosphorylating MDM2 at specific serine residues (166, 183, or 188) to facilitate MDM2 translocation into the nucleus and reduce p53-dependent gene transactivation, and increase the degradation of p53 [65,66,67]. Exemplifying the importance of these residues, in murine HCC models, the generation of mice with Ser 183 replaced by alanine in MDM2 reduced HCC load, suggesting that the absence of Ser 183 phosphorylation sensitizes cells to oxidative stress-induced senescence and ultimately HCC progression [68].

In the other direction, p53 can induce genes that restrict PI3K/Akt/mTOR activity. p53 can induce insulin-like growth factor binding protein 3 (IGF-BP3) which binds to insulin growth factor-1 (IGF1) and prevent activation of the IGF receptor and PI3K/Akt signaling. The tumor suppressor PTEN can also be upregulated by p53 to limit PI3K/Akt signaling [69,70]. p53 can also promote the expression of Sestrins 1/2 that bind the major metabolic regulator adenosine monophosphate-activated kinase (AMPK) and TSC1/2 leading to GAP inhibitor activity and reduced mTOR function [71]. Additionally, p53 increases the expression of regulated in DNA damage 1 (REDD1) which disrupts the inhibitory association of TSC2 and 14-3-3, causing the release of TSC2 and subsequent mTORC1 inhibition [72,73]. The PI3K/Akt pathway plays an important role in activating glycolysis by stimulating the translocation of glucose transporters (GLUT) to promote glucose uptake and activate glycolytic enzymes. Conversely, stabilized p53 can reduce the activity of the pentose phosphate pathway and promote glutaminolysis and mitochondrial oxidative phosphorylation. Thus, increased, mutated, or absent p53 can have profound effects on PI3K/Ak/mTOR pathway activity [74]. In this regard, a recent study illustrated that TSC1 deficiency facilitates p53 haploinsufficiency-mediated activation of PTEN/Akt/mTOR axis to promote HCC tumorigenesis and metastasis [75].

### 4.6. Nuclear Factor Kappa-Light-Chain-Enhancer of Activated B Cells (NF-κB)

PI3K/Akt can regulate the NF-κB pathway/family of transcription factors that modulate inflammation, cellular stress, and innate and adaptive immune responses, which, in turn, regulate the survival, proliferation, migration, and invasion of hepatocytes, kupffer cells, and hepatic stellate cells [76]. NF-κB comprises five different family members: RelA (p65), RelB, c-Rel, p105/p50, and p100/p52. The Rel proteins are synthesized as mature proteins, and p50 and p52 are generated by proteosomal degradation of precursor forms p105 and p100, respectively. The proteins share a conserved Rel homology domain, that binds a target DNA sequence, for homo/heterodimerization, and in the absence of any stimulus are localized to the cytoplasm by inhibitor of NF-κB (IκB) [77]. Activation of NF-κB occurs through distinct canonical and non-canonical pathways. In the canonical pathway, the inhibitor IκB is phosphorylated by the IκB kinase (IKK) complex and subjected to ubiquitin-mediated proteasomal degradation. The free NF-κB dimers (mainly p50/p65 and p50/c-Rel) then translocate to the nucleus and activate target gene transcription. In the non-canonical pathway, the NF-κB-inducing kinase (NIK) is activated, followed by subsequent post-translational processing of p100 into the p52 subunit, dimerization with RelB, and nuclear localization to cause the induction of gene transcription [78].

The PI3K/Akt/mTOR pathway activates the NF-κB pathway through various mechanisms. The most common is the phosphorylation of IKK and IκB by Akt causing dissociation of IκB from the NF-κB dimers [79]. Akt can also promote IKK activity indirectly through mTOR and the mitogen-activated protein kinase (MAP3K) cancer osaka thyroid (Cot) [80,81]. Given that HCC most often develops in the setting of chronic inflammation, the NF-κB pathway has been demonstrated to be a major contributor to HCC. Numerous in vitro studies have established that the NF-κB signaling pathway is aberrantly expressed and activated in human HCC cell lines. Functional studies have shown that the overexpression of IκBα in Hep3B cells can lead to an 80% decrease in cell invasion and the suppression of downstream effectors associated with invasion [82]. In murine models, the chemical carcinogen diethyl nitrosamine (DEN) has often been used to induce HCC, which is similar to human HCC development, and to test the role of the NF-κB pathway. The hepatocyte deletion of IKKβ, a subunit of IKK, prevented NF-κB activation and promoted hepatocyte death, but through inflammatory cell action enhanced ROS production, hepatocyte survival, and HCC promotion. In contrast, the absence of IKKβ in both hepatocytes and Kupffer cells decreased HCC progression [83]. Together, these data show that IKKβ regulates inflammatory crosstalk between the hepatocytes and immune cells, and that aberrant PI3K/Akt/mTOR signaling could drive these events and HCC progression.

### 4.7. Lipid Metabolism

Increased lipid synthesis is required to generate membranes for new tumor cells. Thus, a prominent metabolic change in cancer is to increase the synthesis and uptake of lipids to support cellular growth, proliferation, and tumorigenesis. The PI3K/Akt/mTOR pathway enhances lipid synthesis through different mechanisms. The most relevant is the transcription factor sterol regulatory element-binding protein-1 (SREBP-1), which is a master regulator of lipid homeostasis as it regulates the expression of genes associated with fatty acid, triglyceride, and cholesterol synthesis [84]. SREBP-1 is synthesized in the endoplasmic reticulum (ER) as a precursor protein and to attain nuclear transcriptional activity, the amino domain of SREBP-1 must be released from the ER proteolytically. The processing of SREBP-1 into its mature and active form is influenced by PI3K/Akt/mTORC1 activity, leading to the expression of key lipogenic enzymes, such as fatty acid synthase (FASN), acetyl CoA carboxylase (ACC), and ATP citrate lysate (ACLY) [85,86]. Importantly, studies reveal that SREBP-1 expression is significantly higher in HCC tissues, and this correlates with larger tumor size, higher histological grade, and advanced tumor-node-metastasis stage [87]. Moreover, the suppression of SREBP-1 inhibits HepG2 cell proliferation, migration, and invasion, and in vivo inhibits HCC growth [88].

An important mechanism to regulate SREBP-1 activity involves the phosphatidate phosphatase Lipin 1, which catalyzes the last step of triglyceride synthesis to dephosphorylate phosphatidic acid to generate diacylglycerol, which on mTORC1 phosphorylation becomes localized to the cytoplasm. In the absence of mTORC1 activity, Lipin 1 is dephosphorylated and enters the nucleus and reduces SREBP-transcriptional activity [89]. mTORC1 can also promote lipogenesis through S6K1-mediated phosphorylation of serine arginine protein kinase (SRPK2), causing nuclear translocation and eventual activation of U1 small nuclear ribonucleoprotein 70 kDa (U1-70K) to induce splicing of lipogenic pre-mRNAs, such as FASN, acyl-CoA synthetase long chain family member 1 (ACYL), acyl-CoA synthetase short chain member 2 (ACSS2), and 3-hydroxy-3-methylglutaryl-CoA synthase 1 (HMGCS1), that are important in lipid and cholesterol synthesis [90].

As discussed previously, Akt can inhibit GSK-3 by specific phosphorylation and studies reveal that GSK-3 inhibition leads to SREBP-1 stabilization, and in the absence of GSK-3 inhibition, GSK-3 phosphorylates SREBP-1 and it is inhibited [91]. Moreover, Akt activity is responsible for limiting the expression of insulin-induced gene 2 (Insig2), a liver specific gene and inhibitor of SREBP-1 [92]. mTORC2 has also been shown to control liver fatty acid synthesis through Akt and SREBP-1, leading to broad changes in liver fat synthesis, including sphingolipids, glycerophospholipids, and cardiolipins, with the last of those having the ability to bind ATP synthase and improve oxidative respiration. Importantly, inhibition of mTORC2 reduces HCC fat content and tumor load, and suggests that inhibiting mTORC2 could be a strategy to restrict HCC [93].

### 4.8. Autophagy

Autophagy is a cellular catabolic pathway that targets the degradation or removal of unnecessary, dysfunctional, and long-lived proteins and cellular components for lysosomal degradation. This occurs by the unwanted cellular components being packaged into autophagosomes, a double membrane vesicle that fuses with lysosomes, that then leads to the generation of free fatty acids and amino acids that can be recycled to maintain cellular homeostasis [94]. Autophagy is mediated through various autophagy-related proteins (ATGs) that assemble into several complexes to direct the collection, formation of autophagosome, fusion with lysosome, and eventual degradation [95]. The Akt/mTOR pathway is major regulator of autophagy, and under normal physiological conditions when mTORC1 is activated, autophagy is inhibited through the phosphorylation of ATG1 human homologs Unc-51-like autophagy-activating kinase-1 (ULK1) and Unc-51-like autophagy-activating kinase-2 (ULK2) [96,97]. In conditions of hypoxia or starvation, AMPK is activated and phosphorylates Raptor to inhibit mTORC1 leading to the subsequent dephosphorylation of ULK1/2, and the promotion of autophagy [96,98]. In addition, GSK-3β can also phosphorylate and activate ULK1 to initiate autophagy while suppressing the mTOR pathway [99,100].

Given that ULK1 activation initiates autophagy and autophagy induction is exhibited by many tumors, elevated ULK1 expression is a feature of human HCC and is associated with tumor size and reduced survival time [101]. Functionally, the silencing and deletion of ULK1 in HepG2 and primary human cells suppressed tumor cell proliferation and increased the therapeutic effects of sorafenib in vitro and in vivo. Moreover, the generation of a specific ULK1 inhibitor XST-14 and used in combination with Sorafenib significantly reduces HepG2 tumor growth [102]. Taken together, these data suggest that targeting ULK1 could be a therapeutic approach for treating HCC.

**Figure 1:**
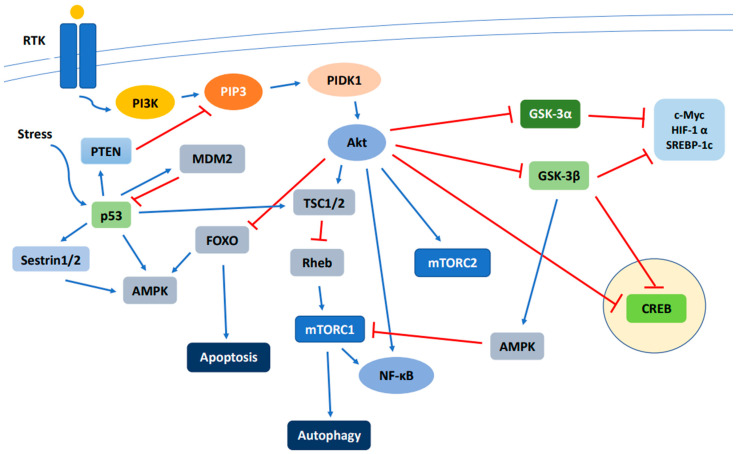
Schematic overview of the major components of the PI3K/Akt/mTOR signaling pathway. Activation of RTK by a ligand in turn activates PI3K, PDK1, and Akt, which regulates downstream effectors including FOXO, mTORC1, mTORC2, CREB, GSK-3α, and GSK-3β. Abbreviations: RTK—receptor tyrosine kinase; PI3K—phosphatidylinositol 3-kinase; PIP3—phosphatidylinositol-3,4,5-triphosphate; PDK1—3-phosphoinositide dependent protein kinase 1; Akt—Akt, protein kinase B; TSC1/2—tuberous sclerosis complex subunit 1/2; Rheb—Ras homolog mTORC1 binding; mTOR—mammalian target of rapamycin; GSK-3—glycogen synthase kinase-3; c-Myc—cellular Myc; HIF-1α—hypoxia inducible transcription factors 1α; SREBP-1c—sterol regulatory element binding protein-1c; CREB—cAMP-response element-binding protein; FOXO—forkhead box O protein; AMPK—adenosine monophosphate-activated protein kinase; MDM2—murine double minute 2; NF-κB—nuclear factor kappa-light-chain-enhancer of activated B cells; PTEN—phosphatase and tensin homolog. Blue line: positive regulation; Red line: negative regulation.

Notwithstanding the aforementioned signaling mediated by the PI3K/Akt/MTOR pathway, it must be mentioned that this pathway is not a stand-alone entity and has cross talk with and can be regulated by the Ras/mitogen-activation protein kinase (MEK)/extracellular signal-regulated kinase (ERK) pathway. The two pathways interact at various signaling points; for example, Akt and mTOR (see review [103]) can be activated by alike growth factors and their concurrent dysregulation is a feature of some HCCs [104]. For further information, we refer readers to recent reviews on the role of Ras/MEK/ERK pathway in HCC [105,106]. The major components of the PI3K/Akt/MTOR pathway as discussed in the text are presented in Figure 1.

## 5. The Utility of PI3K/Akt/mTOR Inhibitors in HCC Treatment

Given that the PI3K/Akt/mTOR pathway is aberrantly activated in near 50% of HCC patients, it is a focus of intense investigation for clinical utility with specific small molecule inhibitors and humanized antibodies to restrict HCC growth. In 2008, the Sorafenib HCC Assessment Randomized Protocol (SHARP) trial (NCT00105443, FDA approval (FDAA) 22 November 2013), a phase III randomized placebo-controlled trial, demonstrated that Sorafenib prolonged median survival and radiologic progression by approximately 3 months in advanced HCC patients (HR 0.69; 95% CI 0.55–0.87; *p* < 0.001) [13]. The results of the SHARP trial led to the approval of sorafenib for HCC treatment by the US Food and Drug Administration (FDA) as the first systemic therapy option for inoperable advanced HCC. In 2009, another randomized placebo-controlled trial known as the Asian-Pacific (AP) trial (NCT00492752) also reported improved overall survival (OS) of 2.3 months (HR 0.68; 95% CI 0.50–0.94; *p* = 0.014), supporting the efficacy of sorafenib in advanced HCC [107]. The REFLECT trial (NCT01761266, FDAA 16 August 2018), a randomized phase III trial, compared lenvatinib and sorafenib as a first line treatment option in advanced HCC patients. This trial demonstrated that the efficacy and safety of lenvatinib is similar to sorafenib (HR 0.92; 95% CI 0.76–1.06) and gained US FDA approval in 2018 as the second first-line systemic treatment option for advanced HCC [108]. Taken together, these three large, randomized trials established the use of sorafenib and lenvatinib to treat advanced HCC and led to the development of numerous clinical guidelines for their use as first line treatment options.

### 5.1. Second-Line Treatment Options

For second-line treatment options, regorafenib is for HCC patients who progressed on sorafenib treatment. A randomized phase III trial (RESORCE; NCT01774344, FDAA 27 April 2017) demonstrated increased median OS of 10.6 months versus 7.8 months for the placebo group (HR 0.63; 95% CI 0.50–0.79; *p* < 0.0001) [109]. Together, this showed that regorafenib provided clinical benefit to sorafenib-resistant patients. A follow-up analysis of the RESORCE clinical trial illustrated a prolongation in time from the beginning of sorafenib treatment to death of 26 months for regorafenib versus 19.2 months for placebo [110].

Cabozantinib is an oral multikinase inhibitor that is often used as a second line or add-on drug. It targets receptor tyrosine kinases such as c-MET, VEGRF2, tyrosine-protein kinase receptor UFO (AXL), and RET to inhibit angiogenesis and Akt activation. Similar to the RESORCE trial, the CELESTIAL trial (NCT01908426, FDAA 14 January 2019) compared the efficacy of cabozantinib versus placebo, and targeted advanced HCC patients with prior sorafenib treatment. The mean OS with cabozantinib was 10.2 versus 8.0 months for placebo (HR 0.76; 95% CI 0.63–0.92; *p* = 0.0005) and the median progression-free survival (PFS) was 5.2 versus 1.9 months (HR 0.44; 95% CI 0.36–0.52; *p* < 0.0001) for cabozantinib compared to placebo [111]. These results, despite a rate of high-grade adverse events, established the effectiveness of cabozantinib as a second-line treatment option for advanced HCC.

In the last decade, humanized antibodies for immunotherapy have been developed to block the interaction of programmed death ligand (PD-L1), which is expressed by tumor cells, with programmed cell death protein 1 (PD-1) present on immune cells, such as T-cells. The purpose of immunotherapies is to block the inhibitory signals mediated between PD-L1 and PD-1 and to promote an anti-tumor response. In 2020, the US FDA approved atezolizumab (a PD-L1 inhibitor) in combination with bevacizumab (a humanized antibody that targets VEGF) as a first-line systemic treatment option for advanced HCC patients without prior systemic treatment [112]. This was based on IMBrave150 (NCT03434379, FDDA 29 May 2020), a randomized phase III study that compared the combination of atezolizumab and bevacizumab versus sorafenib monotherapy. The combination of atezolizumab and bevacizumab increased OS by nearly 6 months (HR 0.66; 95% CI 0.52–0.85; *p* = 0.0009) and PFS by 2 months (HR 0.59; 95% CI 0.47–0.76; *p* < 0.001) compared to sorafenib monotherapy [113]. These results illustrated a significantly better OS and PFS for advanced HCC patients receiving atezolizumab and bevacizumab versus sorafenib alone. The results of the current significant systemic treatment options are presented in Table 1.

### 5.2. Clinical Trials Focusing on the PI3K/Akt/mTOR Pathway

Currently, there are more than 35 ongoing clinical trials targeting the PI3K/Akt/mTOR pathway, and some of the recently completed and ongoing ones we now present. The MATCH Screening Trial (NCT02465060, ongoing) is a phase II clinical trial which will examine the effects of combinations of various inhibitors including sapanisertib (mTORC1/2 inhibitor), GSK2636771 (PI3Kβ inhibitor), capivasertib (ATP-competitive pan-Akt inhibitor), and ipatasertib (Akt inhibitor) in HCC and other cancers. This trial is currently recruiting patients and aims to target advanced tumor patients with no existing-treatment or at least one failed line of standard treatment. For PI3K-targeted clinical trials, SF1126, a dual inhibitor of pan-PI3K and the bromodomain-containing protein 4 (BRD4), an important epigenetic reader, is under a phase I clinical trial for patients with advanced HCC in conjunction with nivolumab (PD-1 immune checkpoint inhibitor; NCT03059147, ongoing) [114].

Nivolumab has been approved as a first-line monotherapy treatment of sorafenib-exposed advanced HCC patients. This was based on the results from the CheckMate040 (NCT01658878, FDAA 22 September 2017) and CheckMate459 (NCT02576509, not approved 30 April 2021) expansion trial. The CheckMate040 trial was a phase I/II trial that evaluated the safety and efficacy of Nivolumab. The CheckMate459 trial was a randomized multicenter phase III study and compared the efficacy of nivolumab in comparison to sorafenib to treat advanced HCC [115]. In comparison to sorafenib, nivolumab showed a median OS during the dose-escalation phase of 15.0 months (95% CI 9.6–20.2) and median time to progression of 3.4 months (95% CI 1.6–6.9) [115]. The CheckmateMate459 results were not promising as median OS was 16.4 months for nivolumab versus 14.7 months for sorafenib (HR 0.85; 95% CI 0.72–1.02; *p* = 0.0752) [116]. In July 2021, nivolumab was voluntarily withdrawn from the market as it failed to meet the requirements for demonstrating benefit in advanced HCC patients. This decision now leaves advanced HCC patients with just three first-line systemic therapies: sorafenib, lenvatinib, and the combination of atezolizumab and bevacizumab.

Given that the clinical trial of atezolizumab and bevacizumab combination was successful, the combination of bevacizumab and erlotinib (EGFR inhibitor) has been explored for its efficacy and safety in an open label phase II trial (NCT01180959, completed 19 May 2021). Erlotinib can prevent activation of Akt signaling pathway. The median OS was 8.55 months in both the bevacizumab and erlotinib combination and sorafenib control group (HR 0.67; 95% CI 0.42–1.07; *p* = 0.09), suggesting no difference in efficacy for this combination and sorafenib monotherapy [117]. Another Akt inhibitor, MK2206 has been considered in two similar clinical trials, but one trial is not specifically for HCC (NCT01425879, ongoing) and the other has been terminated early for discouraging results (NCT01239355, completed 1 February 2013).

Unlike Akt and PI3K inhibitors, mTOR inhibitors have been a more popular target of treatment. Rapalogs, the first generation of mTOR inhibitors, are derived from the antibiotic rapamycin and include everolimus (RAD001), temsirolimus (CCI-779), and sirolimus (rapamycin). Rapalogs are effective in inhibiting HCC cell growth and proliferation as they inhibit phosphorylation of mTOR in vitro and in vivo [118]. Studies examined everolimus as a monotherapy and in second-line combinations in multiple clinical trials. When everolimus was given at 5 or 10 mg/day as a monotherapy, the OS was 8.4 months and only 8% of patients were progression-free at 24 weeks (NCT00516165, completed 1 November 2011). EVOLVE-1 (NCT01035229, completed 1 October 2013), a randomized, double-blind, phase III clinical trial, tested the efficacy of everolimus in sorafenib-failed advanced HCC patients. However, in this study, everolimus was not effective, as OS was 7.6 months with everolimus versus 7.3 months with placebo (HR 1.05; 95% CI 0.86–1.27; *p* = 0.68) [119].

In a randomized phase II clinical trial that compared the efficacy of sorafenib with or without everolimus (NCT01005199, completed 1 March 2016), the median OS was 10 months for sorafenib and 12 months for sorafenib with everolimus, and PFS at 12 months was 70% and 68%, respectively [120]. These results suggest everolimus is not effective in vivo, while as a monotherapy or an add-on there was no prolongation in overall survival. Additionally, another clinical trial that considered the combination of everolimus and MM-141, a monoclonal antibody that targets the insulin-like growth factor 1 receptor (IGF-1R) has been completed, but no result has yet been provided. Other combinations such as bevacizumab and everolimus (NCT00775073, completed 1 April 2012) have been completed and anti-vascular activity has been demonstrated [121]. A clinical trial combining everolimus, trametinib (an inhibitor of the mitogen-activation protein kinase: MEK), and lenvatinib (NCT04803318, ongoing) is active and currently recruiting advanced solid tumor patients, including those with advanced HCC.

Temsirolimus and sirolimus are mTORC1 inhibitors and there are multiple clinical trials associated with both due to their limited efficacy as monotherapies in advanced HCC. A phase I trial of sirolimus combined with bevacizumab (NCT00467194, completed 1 May 2011) is complete with evidence of anti-vascular activity and promising clinical activity [121]. A phase I and phase I/II study of temsirolimus in combination with sorafenib is complete, and the median OS was 8.8 months (95% CI 6.8–14.8) whereas the previous SHARP and Asian-Pacific studies showed 10.7 and 6.5 months, respectively, and this combination has not become an FDA approved treatment option (NCT01008917, completed 27 June 2013; NCT01687673, completed 9 January 2020). Another phase II trial evaluated the combination of temsirolimus and bevacizumab in advanced cancer patients (NCT01010126, completed 13 March 2017) and demonstrated median PFS of 7 months and OS of 14 months, a significant extension of OS, but as the sample size was small (*n* = 28), further studies are required [122]. Furthermore, two active trials are currently recruiting advanced solid tumor patients to evaluate the efficacy and safety of different combinations of mTOR inhibitors, which include nivolumab and nab-rapamycin (ABI-009; a nanoparticle form of human albumin bound rapamycin) (NCT03190174, ongoing); and trametinib combined with everolimus and lenvatnib (NCT04803318, ongoing). Recently, second generation mTOR inhibitors that are under development have begun to be trialed. A phase I/II clinical trial to assess the safety and efficacy of CC-223, a dual mTOR inhibitor, in advanced solid tumor has been completed, but no results are available (NCT01177397, completed 09 December 2016). Furthermore, two clinical trials are actively recruiting patients to evaluate the safety and efficacy of onatasertib (ATG-008), a dual mTOR inhibitor, for both advanced solid tumors and HBV-positive advanced HCC (NCT04518137, ongoing; NCT03591965, ongoing). Table 2 lists ongoing clinical trials with inhibitors targeting the PI3K/Akt/mTOR signaling pathway in HCC.

## 6. Conclusions

Cumulative evidence illustrates the importance of the PI3K/Akt/mTOR signaling pathway in tumorigenesis and HCC progression. The PI3K/Akt/mTOR pathway is overexpressed in nearly 50% of HCCs and the dysregulated activation of this pathway affects a wide range of processes, including cell proliferation, metabolism, tumor cell differentiation, lipid metabolism, autophagy, and EMT [123,124]. Due to these mechanistic connections, the current available first-line drugs are multitargeted tyrosine kinase inhibitors that focus on the PI3K/Akt/mTOR pathway. However, these inhibitors can only modestly extend life; the patients invariably acquire resistance and this in turn leads to limited pharmaceutical options. Thus, with the expected growth in HCC cases worldwide, there is an urgency for the development of safer and more efficient treatment options for advanced HCC patients who are not eligible for surgical resection. In this manner, a key challenge for PI3K/Akt/mTOR pathway inhibition will be to understand the cross-talk and feedback into other pathways and establish methods to diagnose these significant adaptive signaling changes. This could involve more invasive management before and after treatment to determine the basis of each patient’s tumor genetics and signaling changes, the development of patient spheriod screening for drug testing, or the utilization of novel liquid biopsy techniques. Additionally, as is evident from the listed clinical trials, combinations of systemic therapies need to be tested to establish systemic effects and efficacy, develop better inhibitors, and use novel delivery technologies to direct the drug to the tumor. Together, these future developments will reduce side effects and improve life expectancy.

## Figures and Tables

**Table 1:** Summary of significant clinical trials for current first-line systemic treatment options of advanced HCC.

Trial	Drugs	Results (Months)	Statistics	Clinical Trial#
SHARP	Sorafenib vs. placebo	OS 10.7 vs. 7.9	HR 0.69; 95% CI 0.55–0.87; *p* < 0.001	NCT00105443
		SP 4.1 vs. 4.9		
		RP 5.5 vs. 2.8		
Asian-Pacific	Sorafenib vs. placebo	OS 6.5 vs. 4.2	HR 0.68; 95% CI 0.50–0.94; *p* = 0.014	NCT00492752
		TTP 2.8 vs. 1.4	HR 0.57; 95% CI 0.42–0.79; *p* = 0.00005	
REFLECT	Lenvatinib vs. sorafenib	OS 13.6 vs. 12.3	HR 0.92; 95% CI 0.76–1.06	NCT01761266
RESORCE	Regorafenib vs. placebo	OS 10.6 vs. 7.8	HR 0.63; 95% CI 0.50–0.79; *p* < 0.0001	NCT01774344
CELESTIAL	Cabozantinib vs. placebo	OS 10.2 vs. 8.0	HR 0.76; 95% CI 0.63–0.92; *p* = 0.005	NCT01908426
		PFS 5.2 vs. 1.9	HR 0.44; 95% CI 0.36–0.52; *p* < 0.001	
IMbrave150	Atezolizumab + bevacizumab vs. sorafenib	OS 19.2 vs. 13.4	HR 0.66; 95% CI 0.52–0.85; *p* = 0.0009	NCT03434379
		PFS 6.8 vs. 4.3	HR 0.59; 95% CI 0.47–0.76; *p* < 0.001	

The details of completed clinical trials were obtained from https://www.clinicaltrials.gov (accessed on 8 September 2021) and corresponding publications. OS—overall survival; SP—time to symptomatic progression; RP—time to radiologic progression; TTP—time to progress; HR—hazard ratio, CI—confidence interval.

**Table 2:** Ongoing clinical trials with pharmaceuticals associated with PI3K/Akt/mTOR signaling pathway blockade in HCC.

Drugs	NCT #	Patients	Phase	Combinations	Dosage	Year	Status	Notes
Sapanisertib, GSK2636771, Capivasertib, Ipatasertib	NCT02465060	6452	II	Sapanisertib, GSK2636771, Capivasertib, Ipatasertib	Dosages not specified	2015	Recruiting	Aimed for multiple advanced solid tumors
**PI3K inhibitors**								
SF1126	NCT03059147	14	I	Nivolumab	SF1126 IV 900–1100 mg/m^2^ twice weekly + nivolumab 240 mg IV every 2 weeks	2017	Active, not recruiting	
**Akt inhibitors**								
Erlotinib	NCT01180959	45	II	Bevacizumab	Erlotinib 150 mg PO OD + bevacizumab IV 10 mg/kg once every 2 weeks	2010	Completed, no results	As second-line therapy for those with previous sorafenib treatment
MK2206	NCT01425879	8	II		Dosage not specified, but PO every 7 days	2011	Completed	Not specifically for HCC patients—unknown number of HCC patients
MK2206	NCT01239355	15	II		Dosage not specified, but PO every 7 days	2010	Terminated	Early termination due to discouraging results
**mTOR inhibitors**								
RAD001	NCT00516165	28	I/II	RAD001	10 mg PO OD	2007	Completed	P/E and blood test each week and imaging every 6–12 weeks
RAD001	NCT00775073	33	III	Bevacizumab	Everolimus 5 mg PO OD, bevacizumab 5 mg/kg IV every 2 weeks	2008	Completed, no results	
Everolimus	NCT01035229	546	III	Everolimus	7.5 mg PO OD	2009	Completed	
Everolimus	NCT01005199	106	III	Sorafenib	Everolimus 5 mg PO OD, sorafenib 800 mg PO OD	2009	Completed, no results	
Everolimus	NCT04803318	100	III	Trametinib, lenvatinib	PO, dosage not specified	2021	Recruiting	Not specific for HCC, for recurrent/refractory advanced solid tumors
Sirolimus	NCT00467194	27	I	Bevacizumab	Sirolimus 1 mg PO OD, bevacizumab IV 100 mg every 2 weeks	2007	Completed, no results	
Temsirolimus	NCT01008917	25	I	Sorafenib	Temsirolimus IV weekly, sorafenib PO OD	2009	Completed, no results	Combined phase I/II study
Temsirolimus	NCT01687673	29	II	Sorafenib	Temsirolimus IV 10 mg weekly, sorafenib 200 mg PO BD	2012	Completed, results available	
Temsirolimus	NCT01010126	252	II	Bevacizumab	Temsirolimus IV weekly, bevacizumab IV every 2 weeks	2009	Completed, results available	Not specific for HCC, for advanced endometrial, ovarian, liver, carcinoid or islet cell cancer
Nab-rapamycin/ ABI-009	NCT03190174	40	I/II	Nivolumab	Escalating dose of ABI-009 IV once every three weeks, nivolumab 3 mg/kg IV every three weeks	2017	Recruiting	Not specific for HCC, for advanced sarcoma and certain cancers
CC-223	NCT01177397	173	I/II		CC-233 dose starting at 7.5 mg PO OD, dose-escalation	2010	Completed, no results	Not specific for HCC, for advanced solid tumors, non-Hodgkin lymphoma or multiple myeloma
ATG-008	NCT04518137	48	II		ATG-008 30 mg PO, OD	2020	Recruiting	Not specific for HCC, for advanced solid tumors
ATG-008	NCT03591965	75	II		ATG-008 45 mg PO OD, ATG-008 20 mg PO BD	2018	Recruiting	Hep B positive HCC patients with prior exposure to systemic therapy

Abbreviations: PO (orally); OD (once daily); IV (intravenous).

## Data Availability

Not applicable.
